# Improving the latency for 5G/B5G based smart healthcare connectivity in rural area

**DOI:** 10.1038/s41598-024-57641-7

**Published:** 2024-03-23

**Authors:** Arun Kumar, Nishant Gaur, Aziz Nanthaamornphong

**Affiliations:** 1 Department of Electronics and Communication Engineering, New Horizon College of Engineering, Bengaluru, India; 2 Department of Physics, JECRC University, Jaipur, India; 3 College of Computing, Prince of Songkla University, Phuket Campus, Phuket, Thailand

**Keywords:** Smart hospital, Latency, 5G, 6G, QRM-MLD, Electrical and electronic engineering, Computer science

## Abstract

Smart hospitals are poised to greatly enhance life quality by offering persistent health monitoring capabilities. Remote healthcare and surgery, which are highly dependent on low latency, have seen a transformative improvement with the advent of 5G technology. This has facilitated a new breed of healthcare services, including monitoring and remote surgical procedures. The enhanced features of 5G, such as Enhanced Mobile Broadband (eMBB) and Ultra-Reliable Low Latency Communications (URLLC), have enabled the development of advanced healthcare systems. These systems reduce the need for direct patient contact in hospitals, which is especially pertinent as 5G becomes more widespread. This research presents novel hybrid detection algorithms, specifically QR decomposition with M-algorithm maximum likelihood-minimum mean square error (QRM-MLD-MMSE) and QRM-MLD-ZF (zero forcing), for use in Massive MIMO (M-MIMO) technology. These methods aim to decrease the latency in MIMO-based Non-Orthogonal Multiple Access (NOMA) waveforms while ensuring optimal bit error rate (BER) performance. We conducted simulations to evaluate parameters like BER and power spectral density (PSD) over Rician and Rayleigh channels using both the proposed hybrid and standard algorithms. The study concludes that our hybrid algorithms significantly enhance BER and PSD with lower complexity, marking a substantial improvement in 5G communication for smart healthcare applications.

## Introduction

Owing to the increase and upgrade of technologies, hospitals are becoming smarter. However, hospitals are still not completely smart owing to several hurdles, such as implementation costs, an effective trained workforce, the various requirements of smart hospitals, and a lack of infrastructure^1^. The implementation of high-standard smart hospitals will provide great benefits to rural areas where better connectivity, good infrastructure, and medical facilities are not yet available. Smart hospitals can offer remote consultations, telemedicine, and access to specialist services that may be unavailable in rural locations. This can significantly reduce travel time and costs for patients, and improve overall access to care. Real-time data collected through smart systems can help optimize resource allocation, improve public health planning, and better address the specific health needs of rural communities. Smart technologies, such as artificial intelligence (AI)-powered diagnostics and robotic surgery can increase the accuracy and efficiency of healthcare delivery, potentially benefiting everyone, including those in rural areas. The benefits of smart hospitals extend beyond those in rural regions. Urban areas can also benefit from improved access to specialized care, enhanced diagnostics, and data-driven decision-making. Additionally, smart hospital initiatives could reduce hospital overcrowding and wait times by utilizing resources effectively and offering remote consultations to ease pressure on urban healthcare systems and personalize healthcare, and smart technologies can help tailor treatment plans and interventions to individual patient needs, regardless of location^2^. Patients from rural areas must travel several miles or days to visit good healthcare providers. However, it is well established that several illnesses can be curbed by sitting at home with proper monitoring by healthcare professionals ^3^. Additionally, some minor surgeries can be performed in rural areas through video conferencing between medical professionals in rural areas and throughout the world. In the present scenario, remote surgery through a video conference in most rural areas is doubtful because of the lack of infrastructure requirements and high latency ^4^. Latency is one of the most important metrics for proper deployment of smart hospitals. The latency offered by 4G is 20–30 ms because real-time video streaming during health care assistance in remote places is not possible ^5^. In recent years, several academicians, researchers, and engineers have focused on and proposed several standards to reduce the latency of the framework ^6^. Until now, there has been no concrete technology that offers low latency in all conditions. The latency of the framework is affected by the enormous amount of traffic originating 24/7, the demography of the region, the population, and so on. Hence, we must make several changes to the physical layer. Hence, the design of an advanced waveform in the physical layer, which offers low latency and is comparable to Multiple Inputs and Multiple Outputs (MIMO), will play a critical role in developing 6G-based smart hospitals ^7^. The design of an advanced detection algorithm for a MIMO system is a complicated task because of the implementation of several antennas at the base station. In recent years, several detection methods such as ZFE ^8,9^, maximum likelihood (ML) ^10–12^, and MMSE ^13–15^, have been implemented and proposed for the 4G/5G framework. However, the proposed defection algorithms always compromise the throughput and complexity of the framework ^16^. QRMLD holds significant importance in various fields, particularly in digital communication and signal processing. Communication systems enable efficient symbol detection in fading channels, where signals are corrupted by noise and interference. QR decomposition separates the channel effects, making it easier to estimate the transmitted symbols accurately. The M-algorithm optimally selects the most likely symbol sequence and minimizes errors. This approach enhances the reliability and efficiency of wireless communication, improves data rates, and reduces bit-error rates. Moreover, it has applications in various fields, such as image processing, finance, and scientific computing, where accurate data recovery is crucial ^17^. In this study, we propose a novel hybrid detection algorithm for the 6G framework that reduces latency and provides optimal throughput with minimal latency. In the global scenario, the migration of people from rural to urban areas is increasing daily, and these are not to see or monitor the health issues faced by elderly people, especially in rural areas. The lack of good healthcare service professionals and infrastructure forced them to travel to towns and cities, which involved additional transportation costs, waiting for several days for appointments, and unaffordable medical treatment ^18^. With the implementation of a smart healthcare system, the best doctors from cities can monitor patients in rural areas. Even remote surgery is possible under the guidance of expert healthcare monitoring from the best hospitals across the world. The 6G radio provides a faster data rate; hence, patients can share their medical reports with the best doctors worldwide and their expert opinions without traveling anywhere. An increase in population cannot be achieved by conventional hospitals. Hence, the use of advanced techniques to transform conventional hospitals into smart healthcare service providers is necessary. In the case of any pandemic structure, smart healthcare infrastructure can provide a suitable way for people to step outside their homes ^19^. The focus of this study is the development of a hybrid detection method that integrates QRM-MLD with beamforming (BF) to optimize latency, spectrum efficiency, and throughput in large-scale MIMO and Non-Orthogonal Multiple Access (NOMA) systems, aiming to leverage the capabilities of 5G networks ^20^. The investigation delves into the application of progressive waveforms, such as NOMA, Universal Filter Multi-Carrier (UFMC), and Filter Bank Multi-Carrier (FBMC), along with the traditional Orthogonal Frequency Division Multiplexing (OFDM) techniques. Key attributes such as power spectrum density (PSD), bit error rate (BER), system capacity, and peak-to-average power ratio (PAPR) are thoroughly examined within this research framework, highlighting their impact on the performance of advanced 5G wireless communication systems ^21^. 5G has improved the delivery of healthcare services such as monitoring, care, and remote surgery. According to the authors’ findings, 5G for smart healthcare is still in the concept and feasibility analysis stage of development. The limited application of smart healthcare systems, as reported, is largely due to their nascent stage of deployment, with only 15.91% (corresponding to seven) of the reviewed studies showcasing operational smart healthcare implementations ^22^. In their analysis, the researchers drew a comparison between 4 and 5G technologies, concluding that 5G offers a tenfold reduction in latency relative to 4G and possesses the capacity to support a substantially higher number of concurrent devices. Furthermore, it discusses leveraging 5G new radio (NR) to deliver improved healthcare facilities online. Additionally, the use of 5G NR makes data exchange and disease diagnosis quicker and simpler ^23^. The need for 5G in healthcare is examined in this study. In our review, we succinctly examined the core characteristics and functional pillars of 5G technology pertinent to healthcare services. We then identify and elaborate on the most significant healthcare applications enabled by 5G. This technology is anticipated to afford users enhanced management of their health. The advent of 5G is expected to usher in innovative medical technologies that empower patients to conduct health tests and monitoring within their homes ^24^. Additionally, the study referenced as ^25^ sheds light on the system architecture emerging from the integration of IoT technology in smart hospital settings, including the optimization challenges, potential barriers, and the available solutions to address them. To do this, the necessary technical infrastructure was divided into five layers, with each layer’s system architecture, limitations, and techniques outlined. This also covers the size of the smart hospital, scope of its intelligent computing, and extent of its real-time big data analytics. The use of these strategies in various smart-hospital applications was examined in this study. Three matrices were used to analyze the performance of each slicing approach in a hospital network: bandwidth usage, handover count, and block count^26^. In Ref.^27^, the authors provided a thorough analysis of IoT-based 5G-supported smart healthcare solutions. By categorizing and classifying current materials, the structure of smart health care in 5G was analyzed. Furthermore, the crucial conditions for the effective deployment of smart healthcare systems in specific 5G scenarios are outlined. Finally, the number of unresolved problems and research impasses in 5G smart-healthcare IoT solutions were analyzed. To identify opportunities, constraints, and obstacles associated with the implementation of 5G and the ecosystem it has created, the significant transformational qualities of 5G communications and other cutting-edge technologies are outlined and compared with healthcare needs. Additionally, the requirements for administrative infrastructure, medical and surgical education and research, and clinical applications are met. The authors also examined the non-technical issues that Favor opposed the latest healthcare makeover^28^. The document introduces the industry multi-access edge platform (iMEP), a cloud platform tailored for smart healthcare, designed to meet the unique needs of hospitals and support their ongoing development. In addition, the authors in reference^29^ suggest a 5G-enabled framework for the foundational smart healthcare information infrastructure. In the meantime, the implementation process and associated field test outcomes are reported, demonstrating the significant network performance improvement brought about by the proposed system structure. The sixth generation (6G) is expected to be crucial for developing healthcare applications. The development of telehealth in sophisticated healthcare applications is discussed in this chapter. This study describes new developments in intelligent medical applications. The chapter in Ref.^30^ also focused on the privacy and security concerns of future healthcare apps. The development of telehealth and smart healthcare applications has accelerated by the arrival of fifth-generation networks. This paper in Ref.^31^ conducts a thorough review of research articles addressing latency in IoT and cloud systems. It identifies key factors such as network topology, device limitations, and protocol choice impacting latency, and analyzes various proposed solutions, including fog computing and edge processing. This article on Ref.^32^ explores latency challenges in the metaverse and proposes a trust and reputation management system to incentivize low-latency service provision and network optimization. The review in Ref.^33^ discusses various latency issues faced in IoT networks and delves into different solution approaches. It covers techniques such as traffic optimization, resource allocation, and edge computing. The review in Ref.^34^ focuses on utilizing delay-tolerant networks (DTNs) to offer Internet connectivity in regions with challenging network conditions. DTNs can tolerate delays and intermittent connectivity, making them suitable for use in rural areas. The article is organized as follows: The introduction and literature review are presented in “Introduction” section, results are presented in “Results” section, the discussion is presented in “Discussion” section, and the Methods and conclusion of the proposed article is presented in “Conclusion” and “Methods” sections. The study’s contributions are outlined as follows:

- We introduce innovative detection algorithms, specifically QRM-MLD-ZFE and QRM-MLD-MMSE, aimed at reducing latency within the NOMA-MIMO framework.
- When compared to existing standard techniques, our proposed algorithms demonstrate superior performance, achieving an SNR improvement of 3.2 dB.
- The paper provides an extensive review of the requisites and challenges associated with implementing a 6G-based smart hospital infrastructure.

## Results

In this section, we use MATLAB 2016 to analyze and estimate the performance of the proposed hybrid algorithm and the conventional methods. The simulation parameters are listed in Table 1.

**Table 1:** Parameters.

Waveform: NOMA
Transmission scheme: 256 QAM 64X64
N-FET: 256
Symbols: 20,000
Sub-Carriers:256 and 64
Channel: Rician Channel

### BER analysis of proposed algorithm for Rician and Rayleigh channel

**Figure 1:**
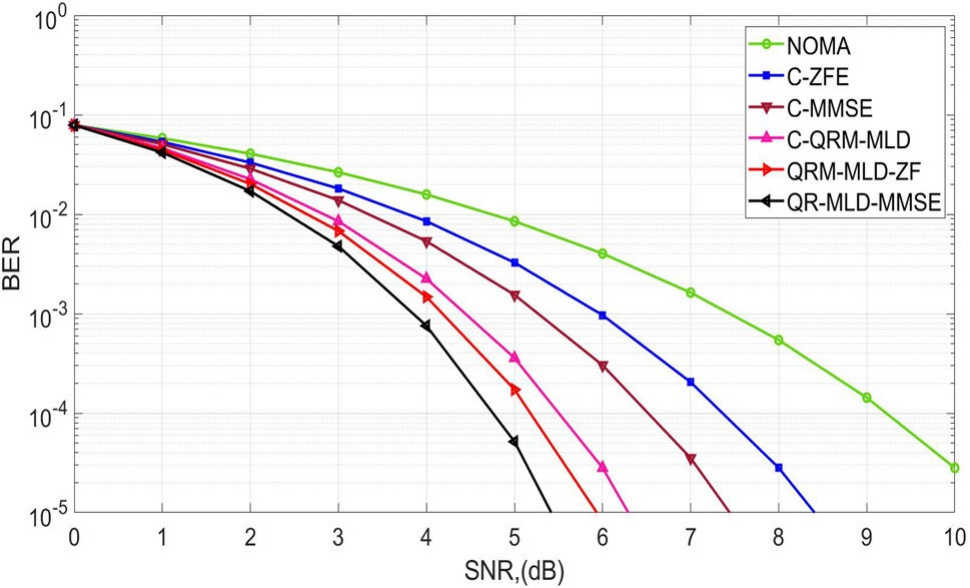
BER performance for 64-sub-carriers in Rician channel.

Figure 1 presents the evaluation of the BER performance for our developed algorithm applied to NOMA waveforms with 64 sub-carriers. The graph compares the BER for various hybrid and conventional detection schemes, including QR-MLD-MMSE, QRM-MLD-ZF, C-QRM-MLD, C-MMSE, and C-ZFE. The results indicate that a BER of 10^−3^ is achieved at an SNR of 3.8 dB with the proposed QRM-MLD-MMSE algorithm, 4.1 dB with QRM-MLD-ZF, 4.7 dB with C-QRM-MLD, 5.6 dB with C-MMSE, and 6 dB with C-ZF. Consequently, the QRM-MLD-MMSE algorithm shows an SNR gain of 0.3 dB, 0.9 dB, 1.8 dB, and 2.2 dB respectively when compared to the conventional methods. It was concluded that the BER performance for 64-sub-carriers in a Rician channel is significant for latency reduction in the communication framework. By achieving a low BER, the proposed hybrid detection methods ensure reliable data transmission and minimize the need for retransmissions or error correction, thus reducing latency and improving overall system responsiveness.

**Figure 2:**
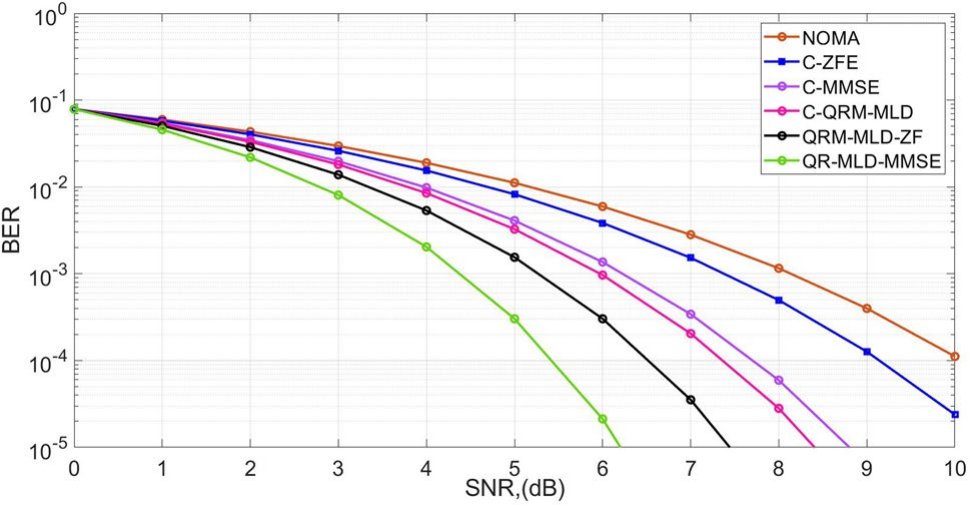
BER performance for 64-sub-carriers in Rayleigh channel.

The BER performance of the NOMA for 256 subcarriers is shown in Fig. 2. The BER for 10^–3^ is obtained at the SNR of 4.8 dB, − 5.2 dB, 6 dB, 6.2 dB, and 7.4 dB by QR-MLD-MMSE, QR-MLD-ZF, C-QR-MLD, C-MMSE, and C-ZFE. The gains obtained by the proposed algorithms are 3.2 dB and 2.8 dB as compared with the NOMA waveform. It is also noted that an increase in the number of sub-carriers reduces the throughput performance of the framework. The BER performance of 64-sub-carriers in Rayleigh channels is crucial for evaluating the reliability of the MIMO framework. This determines the effectiveness of hybrid detection methods in combating fading and noise, guiding the design of robust systems for high-speed data transmission in diverse environments.

**Figure 3:**
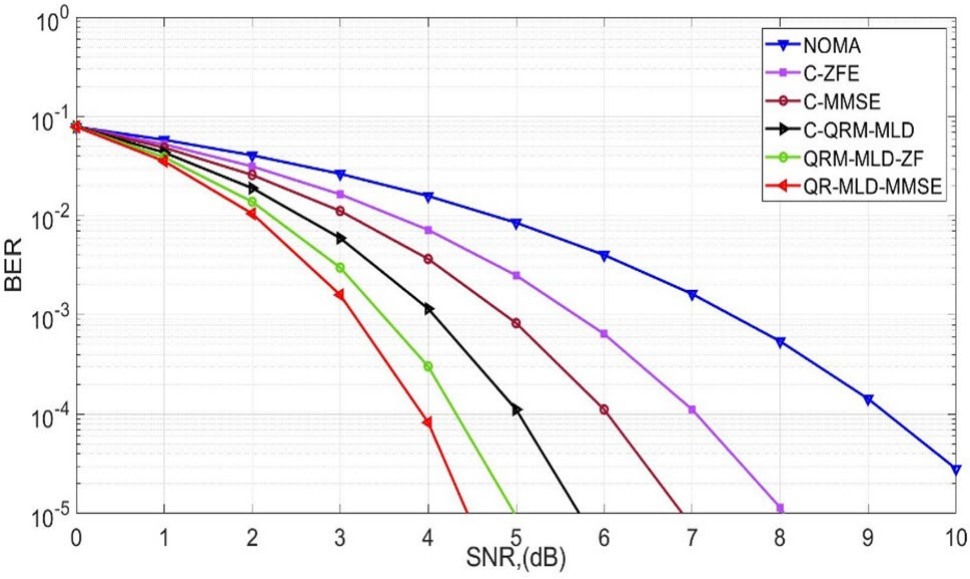
BER performance for 256-sub-carriers in Rician channel.

Figure 3 depicts the Bit Error Rate (BER) performance for both the newly proposed detection algorithms and the conventional methods, as applied to a 64-sub-carrier Rician channel. An initial BER of 10^−3^ is attained at an SNR of 7.2 dB with NOMA prior to the application of the detection methods. The implementation of these algorithms results in a reduction of the required SNR to achieve the same BER, with figures dropping to 3.1 dB, 3.5 dB, 4.1 dB, 5 dB, and 6.1 dB. Therefore, the analysis concludes that the proposed QR-MLD-MMSE algorithm surpasses NOMA by achieving an SNR gain of 4.1 dB. The comparison also reveals that the BER performance of the proposed method is superior on a Rician channel as compared to a Rayleigh channel. Therefore, it is concluded that the 256-sub-carrier BER performance prediction in Rayleigh channels is essential for communication system optimization. To achieve dependable data transmission, it directs the selection of appropriate modulation schemes, coding strategies, and detection algorithms. Reliable performance and optimal spectrum efficiency are ensured by precise bit error rate (BER) estimates, which are necessary to satisfy quality-of-service standards in wireless networks.

**Figure 4:**
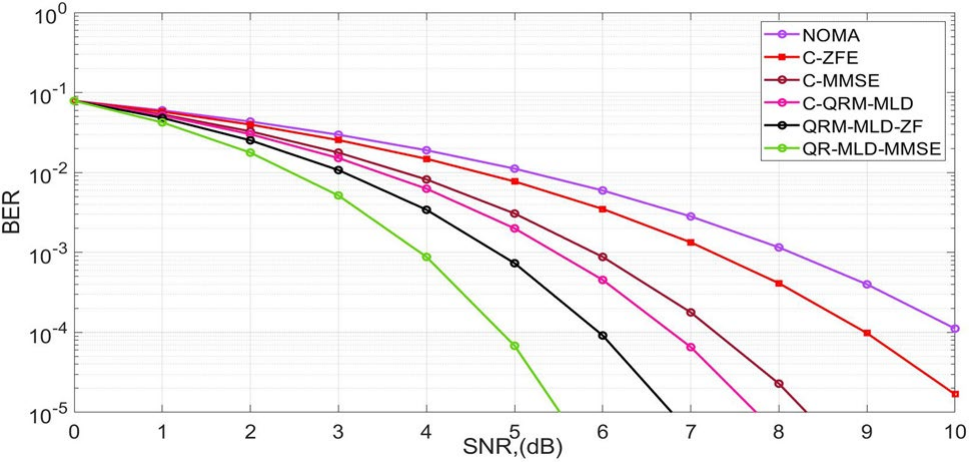
BER performance for 256-sub-carriers in Rayleigh channel.

Figure 4 shows the BER performance of the 256 subcarrier NOMA waveforms under a Rician channel. In this case, the BER gain of 4 dB was obtained by the proposed QR-MLD-MMSE, and the proposed QR-MLD-ZF obtained a gain of 3.2 dB as compared with the NOMA framework. Finally, it was concluded that the BER in 256-sub-carriers in a Rician channel is crucial for signal detection because of its impact on system reliability and performance. This determines the effectiveness of hybrid detection methods in combating fading and interference and guiding the design of robust communication systems. An accurate BER assessment helps optimize detection algorithms, ensuring reliable detection of signals across a wide frequency spectrum, which is essential for maintaining high-quality communication in diverse environments. By enhancing the detection methods and error correction strategies, BER estimation for 256-sub-carriers in Rayleigh channels contributes to the reduction of latency. Reduced retransmissions guarantee improved reliability and reduced packet-processing time. This promotes faster data delivery, lowers the total communication latency, and enhances the real-time responsiveness of wireless networks.

Finally, the comparison of BER performance between 256-sub-carriers and 64-sub-carriers in a Rician channel reveals insights into communication system behavior across different frequency bandwidths. Generally, with 256-sub-carriers, there is a potentially higher spectral efficiency, offering more robustness against frequency-selective fading owing to finer frequency granularity. Consequently, the BER performance in a Rician channel might exhibit better resilience with 256-sub-carriers compared to 64-sub-carriers, particularly in environments with severe multipath or fading effects. However, this results in an increased computational complexity and potentially higher power consumption. Therefore, a tradeoff between spectral efficiency, complexity, and BER performance must be considered based on specific application requirements. Furthermore, a comparison of the BER performance in Rayleigh channels between 64 and 256 subcarriers provides information about the robustness and scalability of the system. Although 256-sub-carriers have a higher spectral efficiency than 64-sub-carriers, they may be more susceptible to noise and fading. Consequently, the BER may be higher. 256-sub-carriers can, however, provide higher throughput and capacity even with higher BER, provided they are equipped with appropriate channel coding and detection algorithms.

### Power spectral density analysis of proposed algorithm for Rician and Rayleigh channel

**Figure 5:**
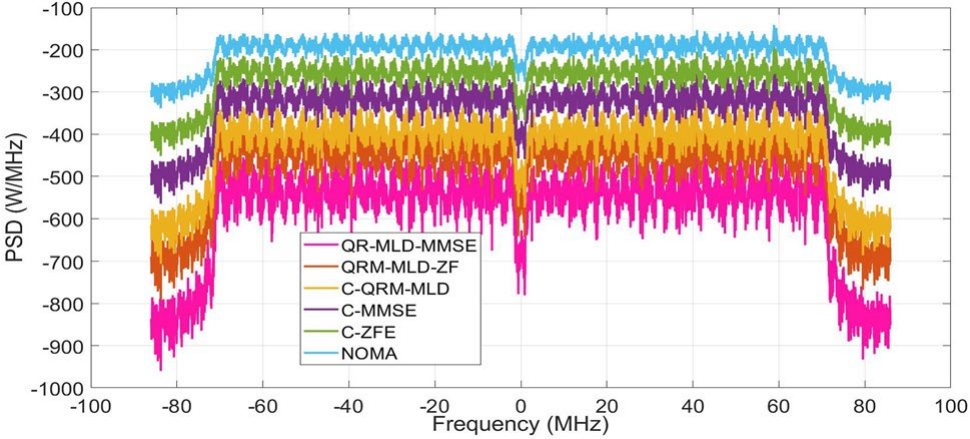
PSD of 256-sub-carriers Rayleigh channel.

In this section, we analyze the out-of-band emission (OOBE) performance of the 256 sub-carrier NOMA under a Rayleigh channel, as shown in Fig. 5. It can be observed that the proposed algorithms and conventional methods efficiently reduce the OOBE of the framework. Power spectral density (PSD) values of − 970, 786, − 670, − 560, − 430, and − 330 were obtained using the proposed QR-MLD-MMSE, QRM-MLD-ZF, C-QRM-MLD, C-MMSE, C-ZFE, and NOMA waveforms. The QR-MLD-MMSE obtained a gain of − 640 compared with NOMA without detection methods. Analyzing the PSD for 256 subcarriers in a Rayleigh channel illuminates the distribution of the signal strength throughout the frequency range, thereby improving the effectiveness of the framework. Gaining an understanding of PSD facilitates more efficient subcarrier allocation and bandwidth optimization. Because the framework can match sub-carrier assignments to channel properties, such as fading, communication systems can have better performance and throughput because they use the spectrum more efficiently.

**Figure 6:**
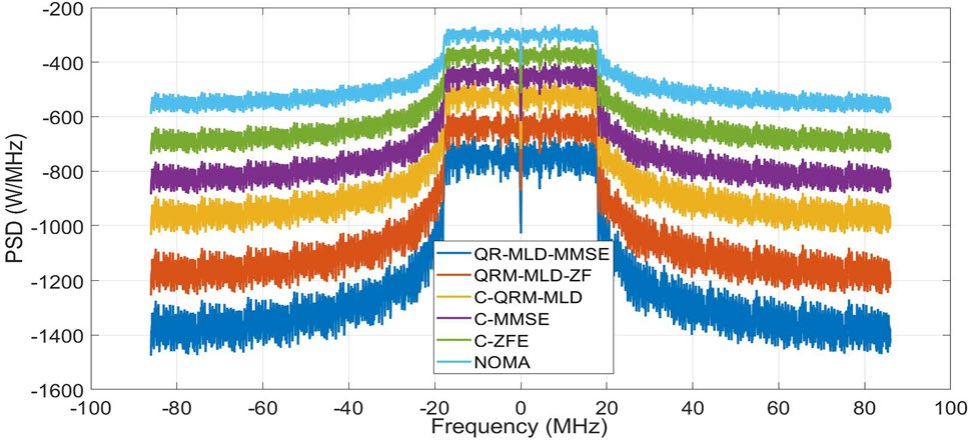
PSD of 256-sub-carriers Rician channel.

Figure 6 shows the PSD performance analysis of the 256-sub-carrier NOMA under a Rician channel. A PSD value of -590 was obtained using the NOMA waveform. It can be observed that the OOBE emission of NOMA is further suppressed by applying the detection methods. However, the proposed QRM-ML-MMSE and QRM-MLD-ZF further enhance the spectral performance by obtaining PSD value of − 1420 and − 1210 as compared with conventional methods such as C-QRM-MLD (− 990), C-MMSE (− 820), and C-ZF (− 670, respectively). Hence, it was concluded that the detection algorithms significantly enhance the spectral performance of the framework. Additionally, it was observed that the NOMA in the Rician channel performed better than that in the Rayleigh channel. For optimal resource allocation and signal transmission in a Rician channel, it is essential to estimate the PSD of the 256 subcarriers. It offers insightful information regarding the properties of the frequency domain, facilitating the effective use of the available bandwidth and reducing interference. In wireless communication networks, precise PSD estimates improve throughput, spectral efficiency, and system performance.

### Complexity analysis

Complexity in signal detection and communication systems refers to the intricacy of algorithms and processes involved in extracting meaningful information from signals amidst noise and interference. The significance lies in achieving robust performance despite the diverse environmental conditions, enhancing reliability, and optimizing resource utilization. Complex systems enable advanced modulation techniques, error correction, and adaptive signal processing, which are crucial for modern wireless networks, radar systems, and digital communications. Balancing complexity is vital for ensuring efficient implementation without sacrificing performance, making it pivotal in enhancing communication reliability, data throughput, and overall system efficiency in increasingly demanding communication environments. In signal detection, complexity refers to the computational resources required to process and decode signals effectively^35^. Because 5G networks use advanced modulation schemes and massive MIMO configurations, the complexity parameter becomes crucial. High complexity can strain mobile devices and base stations, thereby leading to increased power consumption and latency. Balancing the complexity is essential to ensure efficient signal detection and maintain the desired performance while keeping the hardware and energy demands manageable. Advanced algorithms, hardware acceleration, and optimization techniques play a vital role in addressing the complexity challenge of 5G signal detection, enabling the network to deliver high data rates and low latency while conserving resources. Table 2 lists the complexities of the proposed and the conventional algorithms.

**Table 2:** Complexity.

S.no	Algorithms	Complexity
1	MMSE	$O({N}^{3})$
2	Zero-Forcing Equalization	$O({N}^{2} \times M)$
3	QRM-MLD	$O({2}^{N + M})$
4	QRM-MLD-ZF	$O(2^(N + M) )\times {M}^{3}$
5	QRM-MLD-MMSE	$O({N}^{2} \times M)$

## Discussion

Reducing latency in 5G-based smart hospitals is crucial for enabling real-time communication and data transfer for various healthcare applications. Addressing these challenges requires collaboration among healthcare providers, technology vendors, and regulatory bodies to create a seamless and low-latency 5G ecosystem in smart hospitals, ultimately improving patient care and safety. In this work, we deal with only one challenge, that is, improving the latency of the framework by using the proposed QR-MLD-MMSE and QR-MLD-MMSE methods. However, several challenges need to be addressed to achieve high performance in such environments. The listed challenges are very important and further improve the performance of the framework if properly implemented^36^.

- *Network infrastructure* Deployment of 5G infrastructure with adequate coverage and capacity is essential to ensure low-latency connectivity throughout the hospital. This includes installing small cells and optimizing the network design.
- *Interference and signal quality* Interference from other devices and networks can degrade the signal quality and increase latency. Proper spectrum management and interference-mitigation techniques are required.
- *Edge computing* Leveraging edge computing resources closer to devices and sensors can help process data locally, reducing the need to transmit data over long distances to centralized servers.
- *Device compatibility* Ensuring that all medical devices and sensors are compatible with 5G technology and that they support low-latency communication is crucial. Legacy devices may require upgrading or replacement.
- *Security concerns* Implementing robust security measures is vital because low-latency networks can be vulnerable to cyberattacks. Encryption, authentication, and intrusion detection systems are necessary to protect patient data.
- *Quality of service (QoS)* Establishing strict QoS policies to prioritize critical healthcare traffic over noncritical data is essential to maintain low latency for critical applications.
- *Roaming and handovers* Seamless handovers between 5G cells or networks as patients and staff move within the hospital can be challenging. Ensuring minimal disruption during these transitions is crucial in latency-sensitive applications.
- *Resource allocation* Efficient allocation of network resources, such as bandwidth and processing power, is important for preventing congestion and maintaining low latency for all connected devices and services.
- *Redundancy and reliability* Hospitals require high levels of network redundancy and reliability to ensure that critical services are not disrupted. Redundant paths, failover mechanisms, and backup power supplies are required.
- *Regulatory compliance* Meeting regulatory requirements, such as data privacy laws (e.g., HIPAA in the United States), while maintaining low latency can be challenging. Compliance must be the top priority.
- *Cost* Implementing and maintaining a high-performance 5G network in a hospital can be expensive. Hospitals must carefully plan and allocate resources to meet their latency reduction goals.
- *Training and staff adoption* Ensuring that hospital staff are trained to use new technologies and understand the importance of low-latency communication is essential for successful implementation.

The novelties of the proposed article are detailed below, as informed by a comprehensive literature review and the data presented in Table S1^37–43^, to our current understanding.

i. QR-MLD-MMSE combines QR decomposition to reduce the computational complexity with MMSE for better error performance. This hybrid approach can significantly enhance the efficiency of symbol detection, thereby leading to faster processing and reduced latency.
ii. The traditional ML method can be computationally intensive, particularly in massive MIMO scenarios typical of 5G. By incorporating QR decomposition, the complexity of MLD is reduced, enabling faster symbol detection without sacrificing accuracy.
iii. Latency is a critical metric in 5G networks, particularly for real-time applications, such as autonomous vehicles, remote surgery, and industrial automation. QR-MLD-MMSE and QR-MLD-ZF reduce latency by speeding up the detection process, ensuring the timely delivery of data packets and faster response times.
iv. By optimizing the detection process, the proposed QR-MLD-MMSE and QR-MLD-ZF methods improve the spectral efficiency of the communication system. This allows for a more efficient use of the available bandwidth, accommodating higher data rates, and supporting more devices simultaneously without compromising latency requirements

## Conclusion

The upgrade of conventional hospitals to smart hospitals with the help of advanced techniques such as 5G and 6G is a requirement in the present scenario. In this study, we focused on improving the latency of M-MIMO-NOMA to ensure the reliable and efficient real-time transmission of information. It should be noted that the proposed hybrid algorithms obtained efficient BER gains of 4 dB and 3.2 dB compared with conventional frameworks. Furthermore, the OOBE is reduced, resulting in high spectral access to the framework. The complexity of the framework increases with the throughput. However, in this article, the simulation results reveal that high throughput and PSD performance are obtained with trivial intricacy. It was concluded that latency reduction in 5G/Beyond 5G smart healthcare connectivity significantly enhances healthcare services in rural areas. Lower latency ensures real-time telemedicine, enabling patients to consult specialists without travel delays. Remote monitoring of vital signs and chronic conditions becomes more effective as data from medical devices and wearables are transmitted promptly. During emergencies, quick data transfer allows for faster communication with first responders and remote medical guidance. This timely intervention can be lifesaving in rural areas with limited access to immediate medical care. Overall, latency reduction in B5G healthcare connectivity improves healthcare access, quality, and outcomes for rural populations, narrowing the urban–rural healthcare divide. The limitations of the proposed algorithms include complexity scalability issues in large MIMO systems, vulnerability to noise and channel estimation errors, and the inability to fully mitigate ISI and multiuser interference.

## Methods

### QRM-MLD

**Figure 7:**
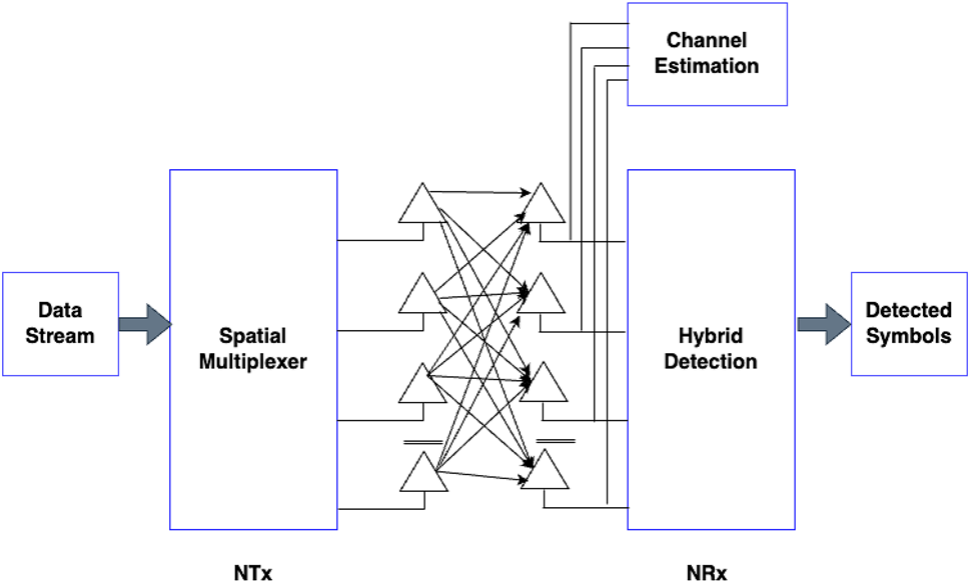
MIMO with hybrid detection.

QR decomposition with the M-algorithm for maximum likelihood detection plays a vital role in reducing latency in 5G networks. In 5G, low latency is critical for real-time applications, such as autonomous vehicles and remote surgery. QR decomposition efficiently mitigates the latency by enhancing the accuracy of symbol detection. Decomposing the channel matrix allows for a more precise estimation of the transmitted symbols even in the presence of interference and noise. This reduces the need for retransmission and reprocessing of data, thereby lowering the communication latency. Consequently, 5G networks benefit from improved reliability and faster data transmission, ensuring that critical applications operate with minimal delay and meeting the stringent latency requirements of emerging technologies^44^. Consider the Massive-MIMO (M-MIMO) system shown in Fig. 7. The received signal in MIMO systems refers to the combined signals received at multiple antennas that exploit spatial diversity for improved communication performance. The received signal in M-MIMO can be represented as

1 $$Z=Hy+{\omega }_{n},$$

where Z is the received signal given by $Z={\lceil{Z}_{1} {Z}_{2}, . . . , {Z}_{m}\rceil}^{T}$, y represents the transmitting signal from ${n}_{th}$ antenna: $y={\lceil{y}_{1} {y}_{2}, . . . , {y}_{n}\rceil}^{T}$, ${\omega }_{n}$ is the noise, and h is the response of the channel given by Ref.^45^.

2 $$H=\left[\begin{array}{ccc}{h}_{\mathrm{1,1}}& \cdots & {h}_{1,m}\\ \vdots & \ddots & \vdots \\ {h}_{n,1}& \cdots & {h}_{n,m}\end{array}\right].$$

The channel response matrix H in MIMO systems is crucial for characterizing wireless channels, enabling efficient beamforming, spatial multiplexing, and diversity techniques for enhanced communication performance and throughput. QRM-MLD involves optimizing the signal detection to maximize the likelihood of the received symbols given the observed data. The conventional QRM-MLD algorithm can be expressed as^46^

3 $$H=QT,$$

where H denotes the channel response, which is split into $n\times n$ unitary matrix (Q) and $m\times m$ triangular matrix (T) given by^47^:

4 $$T=\left[\begin{array}{ccc}{t}_{\mathrm{1,1}}& \cdots & {t}_{1,m}\\ \vdots & \ddots & \vdots \\ 0& \cdots & {t}_{m,m}\end{array}\right].$$

The Eq. (4) in QRM-MLD assists in reducing computational complexity by facilitating efficient relay selection and symbol detection, optimizing performance, while minimizing processing requirements in MIMO systems. In the M-MIMO framework, state $n\le m$ must satisfy to obtain a unique solution. In this equation, we consider $n=m$ and convolve Eq. (1) with Q^H^ as follows:

5 $$\overline{Z }={Q}^{H}\left({H}_{y}+{\omega }_{n}\right)={T}_{y}+\overline{{\omega  }_{n}}.$$

The received signal ($\overline{Z })$ comprises the combined transmissions from multiple antennas, whereas noise ($\overline{{\omega  }_{n}}$) refers to unwanted interference or disturbances affecting the received signal quality. It is noted that the $\overline{Z }$ and $\overline{{\omega  }_{n}}$ are defined as^48^

6 $$\overline{Z }=Z{Q}^{H},$$

7 $$\overline{{\omega  }_{n}}=Z\overline{{\omega  }_{n}},$$

Further, the M algorithm is applied to Eq. (5) to obtain the sequence of transmitting elements: ${\lceil{y}_{1} {y}_{2}, . . . , {y}_{n}\rceil}^{T}.$ Once the sequence of the transmitting element is selected, ML is used to determine the transmitted signal elements. Initially, the selection of the transmitting signal element ${y}_{n}$ was completely demonstrated. ML is determined to obtain a constellation of all the signal elements. Hence, S-time computations are executed as follows^49^:

8 $${\left|\overline{{x }_{n}}-{T}_{n,n}{y}_{n}\right|}^{2}.$$

From Eq. (8), the N nodes that correlate with the minimal N value are adopted, and the rest of the nodes are rejected. The designated node is dilated into $\left|S\right|$ nodes and $N\times \left|S\right|$ computations are executed as follows^50^:

9 $$\bigg\Vert {\left[\begin{array}{c}\overline{{x }_{n-1}}\\ \overline{{x }_{n}}\end{array}\right]-\left[\begin{array}{cc}{t}_{n-1,n-1}& {t}_{n-1,n}\\ 0& {t}_{n,n}\end{array}\right]\left[\begin{array}{c}{y}_{n-1}\\ {y}_{n,s}\end{array}\right]\bigg\Vert }^{2}.$$

The Eq. (9) can be also written as^50^:

10 $${\left|\overline{{x }_{n-1}}-{t}_{n-1,}n-1{y}_{n-1}-{t}_{n-1},n{y}_{n,}S\right|}^{2}+{\left|\overline{{x }_{n}}-{t}_{n,}n{y}_{n,s}\right|}^{2}.$$

Here ${y}_{n,s}$ first selects the contender in the initial step. In addition, the N entrant element is chosen for transmitting signal $\left[{y}_{n-1}{ y}_{n}\right]$, which represents the trivial N parameter from the $N\times \left|S\right|$ values. Likewise, the N entrants signal elements for $\left[{y}_{n-2,}{ y}_{n-1},\dots {y}_{n}\right], . .. \left[{y}_{1},{y}_{2},. . . {y}_{n}\right]$ are considered at every period. Finally, the signal elements $\left[{y}_{1,}{ y}_{2},\dots {y}_{n}\right]$ are determined as the optimal approximated evaluated transmitted signals. The intricacy of the traditional QRM-MLD is less as compared with the ML. However, the prototype implementation requires more computation, leading to high complexity. The QRM-MLD suffers from several drawbacks. Firstly, it's computationally intensive, demanding substantial processing power and time. Secondly, it's sensitive to initialization, potentially leading to convergence to local optima. Additionally, it's prone to numerical instability when dealing with ill-conditioned matrices. To improve detection, one can employ advanced optimization techniques like stochastic approximation or evolutionary algorithms, enhancing convergence and robustness. Furthermore, incorporating regularization techniques can mitigate numerical instability issues. Employing parallel computing can also expedite computation, making it more feasible for real-time applications.

### ZFE

It is considered a simple detection method that is utilized in the M-MIMO framework. The primary aim of the ZFE is to obtain optimal signal detection in the presence of interference and noise. However, prior channel state estimation and noise occur during the ZF process. The ZFE is advantageous for its simplicity and effectiveness in combating inter-symbol interference (ISI) in communication systems. By nullifying interference, it simplifies decoding at the receiver, enhancing signal quality. However, ZFE suffers from noise enhancement, making it susceptible to noise amplification and reducing its performance in noisy channels. In 6G, ZFE proves beneficial due to its low computational complexity, making it suitable for high-throughput and low-latency communications. With 6G’s emphasis on ultra-reliable and low-latency communication (URLLC) and massive connectivity, ZFE can provide efficient equalization, aiding in mitigating interference and improving spectral efficiency. Its simplicity aligns well with 6G’s goal of supporting diverse applications ranging from augmented reality to autonomous vehicles. The ZFE is subject to a few constraints^51^.

We consider an M-MIMO system with antennas at the transmitter and receiver. The M-MIMO system can be represented by Eq. (1):

11 $$Z=Hy+{\omega }_{n}.$$

The ZFE is applied to eliminate the interference between users by designing a linear transformation matrix (M). The matrix neutralizes the interference that occurs from the other user and enhances signal detection. The Rx signal after applying the ZFE can be expressed as follows^52^:

12 $${Z}_{ZFE}=H{y}^{M}+M{\omega }_{n}.$$

The ZFE should be designated such that matrix M satisfies the following condition:

13 $$MH=I,$$

where I denotes the identity matrix. The Eq. (13) ensures that the interference between users is completely eliminated.

### MMSE

The MMSE is considered one of the most popular techniques for efficiently detecting signals in the presence of noise and interference. The MMMSE estimation offers several advantages in signal processing. It provides optimal estimates under Gaussian noise assumptions, effectively minimizing mean square error. MMSE estimation is computationally efficient and simpler to implement compared to other sophisticated techniques. It offers robustness against noise and can handle non-linear systems through iterative algorithms like the Expectation–Maximization (EM) algorithm. However, MMSE estimation relies heavily on accurate knowledge of statistical parameters and assumes linearity in the underlying system, limiting its applicability in highly non-linear scenarios. In the context of 6G, MMSE estimation holds promise due to its ability to effectively mitigate interference and noise, critical in high-density networks. Its computational efficiency makes it suitable for resource-constrained devices expected in 6G networks. Moreover, as 6G aims for ultra-reliable and low-latency communication, MMSE estimation's ability to provide accurate estimates with minimal computational overhead aligns well with these requirements, facilitating efficient spectrum utilization and enhancing overall network performance^53^. Let us consider the mathematical model of M-MIMO represented by Eq. (1).

14 $$Z=Hy+{\omega }_{n}.$$

The goal of MMSE detection is to estimate the transmitted signal y given the received signal with all knowledge of the channel matrix H and noise statistics. The estimation of y, denoted as ŷ is given by^44^:

15 $$\widehat{y}=arg{\text{min}}yE\sum {\left|z-\widehat{z}\right|}^{2},$$

where E denotes the expectation operator. High complexity and prior knowledge of the channel are the few constraints of the MMSE.

### Channel estimation proposed algorithms

Channel estimation is crucial for accurate signal detection in MIMO systems. This enables the receiver to estimate the characteristics of the wireless channel, including fading, interference, and spatial correlations. This information is essential for mitigating the effects of channel impairments, optimizing signal-detection algorithms, and achieving reliable communication performance under diverse environmental conditions. Accurate channel estimation enhances the system’s ability to separate the transmitted signals from noise and interference, leading to improved spectral efficiency, higher data rates, and enhanced overall system reliability in MIMO systems^54^.

### Channel estimation QRM-MLD-ZF

The received signal at the receiver is represented as:

16 $$Z=HY+N.$$

The QR decomposition of channel matrix is performed as:

17 $$H=QR.$$

MLD-ZF precoding aims to mitigate the interference by projecting the received signal onto the null space of the interference channels. The ZF precoding matrix F was computed as follows:

18 $$F={{(H}^{H}H)}^{-1}{ H}^{H},$$

where ${H}^{H}$ is the conjugate transpose of H. Channel matrix H can be estimated using a pilot symbol known at the receiver. Let ${Y}_{p}$ be the transmitted pilot symbols, and ${Z}_{p}$ be the received pilot symbols. Then, the estimated channel matrix $\widetilde{H}$ can be obtained as

19 $$\widetilde{H}={Z}_{p}{Y}_{p}^{H}{\left({Y}_{p}^{H}{Y}_{p}\right)}^{-1}.$$

After estimating channel matrix $\widetilde{H}$, it can be used for symbol detection. The received signal Z can be processed by MLD-ZF precoding as follows:

20 $${Z}^{ZF}=FZ.$$

Then, MLD can be applied to detect the transmitted symbols Y based on the pre-processed received signal ${Z}^{ZF}$. Channel estimation is crucial for accurately estimating channel matrix H, which is essential for performing ZF precoding and subsequent symbol detection in the QRM-MLD-ZF algorithm^55^.

### Channel estimation QRM-MLD-MMSE

Let us consider a MIMO system with ${N}_{t}$ and ${N}_{r}$ transmitting and receiving antennas, respectively. The received signal is given as

21 $$Z=HY+N.$$

The QR decomposition is performed on the received signal matrix $Z$ to obtain:

22 $$Z=QR,$$

where Q is the orthogonal matrix and R is the upper triangular matrix. Substituting Q and R into the channel model, we obtain:

23 $$QR=HY+N.$$

Pre-multiplying both side by ${Q}^{H}$, we have:

24 $${Q}^{H}QR={Q}^{H}HY+{Q}^{H}N.$$

Let us substitute $I={Q}^{H}Q$, where $I$ is the identity matrix and simplifies to

25 $$R={Q}^{H}HY+{Q}^{H}N.$$

The pilot symbols are typically transmitted to estimate the channel matrix H. Let ${Y}_{p}$ be the transmitted pilot symbols, and ${Z}_{p}$ be the received pilot symbols. Then, the estimated channel matrix $\widetilde{H}$ can be obtained as

26 $${\widetilde{H}=(Q}^{H}{Y}_{p}){{(R}_{P})}^{-1},$$

where ${R}_{P}$ covariance matrix of the received pilot symbol ${Z}_{p}$. After estimating channel matrix $\widetilde{H}$ it can be used for symbol detection. The MMSE detection method uses the estimated channel matrix to minimize the mean square error between the transmitted symbol $Y$ and the detected symbol $\widetilde{Y}$, given by

27 $$\widetilde{Y}={\widetilde{H}}^{H}{\left(\widetilde{H}{\widetilde{H}}^{H}+{\sigma }^{2}I\right)}^{-1}Z,$$

where ${\sigma }^{2}$ denotes the noise variance. Channel estimation in QRM-MLD-MMSE involves using QR decomposition to decompose the received signal matrix, estimating the channel matrix using pilot symbols, and then applying MMSE detection for symbol estimation based on the estimated channel matrix. This approach helps improve channel accuracy and symbol detection in the MIMO framework.

### Supplementary Information

Supplementary Information 1.

Supplementary Information 2.

## Data Availability

The algorithms or codes generated and /or analysed during the current study are available from the corresponding author upon reasonable request and permission.

## Code availability

The algorithms or codes generated and/or analysed during the current study are available from the corresponding author upon reasonable request and permission.
