# Supplementary material for: Improving the latency for 5G/B5G based smart healthcare connectivity in rural area
Source: Sci Rep. 2024 Mar 23;14:6976. doi: 10.1038/s41598-024-57641-7 (PMC10960841; doi:10.1038/s41598-024-57641-7)
Supplement: Supplementary file 1 — Supplementary Information 1. [file 41598_2024_57641_MOESM1_ESM.pdf]

Algorithm QRM-MLD-MMSE

```
function [estimated_symbols] = qrm_mld_mmse(received_signal, channel_matrix, transmit_power, noise_variance)
  % QRM-MLD-MMSE algorithm implementation
  % Compute MMSE receiver weights
 mmse_weights = (transmit_power / (transmit_power + noise_variance)) * pinv(channel_matrix);
    % Calculate estimated transmitted symbols
   estimated_symbols = mmse_weights * received_signal;
end
%For the QRM-MLD-MMSE algorithm that incorporates Maximum Likelihood Detection:%
function [final_symbols] = qrm_mld_mmse_with_mld(received_signal, channel_matrix, transmit_power,
noise_variance)
   % QRM-MLD-MMSE with MLD algorithm implementation
   % Compute MMSE receiver weights
   mmse_weights = (transmit_power / (transmit_power + noise_variance)) * pinv(channel_matrix);
   % Calculate estimated transmitted symbols
   estimated_symbols = mmse_weights * received_signal;
   % Maximum Likelihood Detection (MLD)
   % Perform MLD on the estimated symbols to obtain the final detected symbols
   % Placeholder for MLD process
   final_symbols = estimated_symbols; % Replace this line with your MLD implementation
end
```

Algorithm Code for QRM-MLD-ZF

```
function [qrm_symbols, mld_symbols, zf_symbols] = combined_detection(received_signal, channel_matrix)
   % Combined detection using QRM, MLD, and ZF methods
   % QRM detection
   qrm_symbols = qrm_detection(received_signal, channel_matrix);
   % MLD detection
   mld_symbols = mld_detection(received_signal, channel_matrix);
   % ZF detection
   zf_symbols = zf_detection(received_signal, channel_matrix);
end
function [qrm_symbols] = qrm_detection(received_signal, channel_matrix)
 % QRM detection algorithm
   % Placeholder for QRM detection
   % This can be any detection method you want to implement for QRM
   qrm_symbols = received_signal; % Placeholder implementation
end
function [mld_symbols] = mld_detection(received_signal, channel_matrix)
   % MLD detection algorithm
   % Placeholder for MLD detection
   % This can be any detection method you want to implement for MLD
   mld_symbols = received_signal; % Placeholder implementation
end
function [zf_symbols] = zf_detection(received_signal, channel_matrix)
   % ZF detection algorithm
   % Compute the pseudo-inverse of the channel matrix
   pseudo_inverse_channel = pinv(channel_matrix);
  % Perform zero-forcing detection
   zf_symbols = pseudo_inverse_channel * received_signal;
end
```
