# Supplementary material for: Improving the latency for 5G/B5G based smart healthcare connectivity in rural area
Source: Sci Rep. 2024 Mar 23;14:6976. doi: 10.1038/s41598-024-57641-7 (PMC10960841; doi:10.1038/s41598-024-57641-7)
Supplement: Supplementary file 2 — Supplementary Information 2. [file 41598_2024_57641_MOESM2_ESM.docx]

In Table S1, we have compared the proposed work with the related published to analyse the novelty of the proposed work.

**Table. S1: Comparison of the proposed work**

| Reference | Signal detection algorithms |
| --- | --- |
| [11] | System Model: QRM-MLD-BF:  a. Beamforming is a technique that adjusts the phase and amplitude of signals in an array of antennas to focus the transmitted power in a specific direction.  b. In QRM-MLD-BF, the receiver uses beamforming to enhance the received signal quality by aligning the receiver antenna array in the direction of the transmitted signal.  c. While beamforming can improve the signal quality, it may not completely eliminate interference or noise, especially in environments with multipath propagation or interference from other sources.  **Simulations Parameters**: BER is implemented.  Channel: Rayleigh channel  Sub-carriers :64 |
| [35] | System Model: Joint deregularized and box-constrained dichotomous coordinate descent (BOXDCD):  a. The BOXDCD detection algorithm is significant for its ability to efficiently handle high-dimensional data in various applications such as signal processing and machine learning. b. The drawbacks include potential sensitivity to hyperparameters and limited applicability in cases with highly non-linear relationships. Its effectiveness depends on careful parameter tuning and may not perform optimally in all scenarios without appropriate adjustments.  Simulation parameters: only BER is estimated.  Channel: Rayleigh channel |
| [37] | System Model: Maximum Ratio Combining (MRC) with Alamouti Space Time Block Codes (STBC)  a. MRC-STBC detection algorithms are significant for their ability to improve signal reliability in wireless communication systems, particularly in fading channels. MRC optimally combines multiple received signals to enhance the overall signal quality.  b. The drawbacks include increased complexity in implementation and susceptibility to errors in estimating channel parameters, particularly in fast-fading environments. Additionally, MRC may not fully mitigate the effects of severe fading or interference, limiting its effectiveness in certain scenarios.  **Simulation:** BER is implemented for the proposed method.  Channel: Rayleigh channel |
| [38] | System Model: MMSE-alternating direction method of multipliers (ADMM)  a. The MMSE-ADMM detection algorithms are significant for their capability to address the challenging task of signal detection in massive MIMO systems efficiently. They combine the advantages of MMSE estimation and ADMM optimization to achieve robust performance in complex interference scenarios.  b. The drawbacks include sensitivity to parameter settings and computational complexity, especially in large-scale systems. Additionally, convergence issues may arise in certain scenarios, necessitating careful algorithmic design and tuning for optimal performance.  **Simulation:** BER is implemented for the proposed method.  Channel: AWGN channel |
| [39] | System Model: Beamforming Techniques  a. Beamforming techniques are significant in wireless communications for enhancing signal strength and quality. They enable directed transmission and reception, improving coverage, capacity, and reliability in wireless networks. Beamforming can mitigate interference and increase spectral efficiency, crucial for 5G and beyond.  b. The drawbacks include susceptibility to multipath fading, limited coverage area per beam, and complexity in implementation. Additionally, beamforming requires precise channel state information, making it challenging in dynamic environments or when operating in non-line-of-sight conditions.  **Simulation:** No simulations were presented |
| [40] | System Model: zero forcing scheme based on QR matrix  a. The zero-forcing scheme based on QR matrix is significant for its effectiveness in mitigating multi-user interference in MIMO systems. By employing QR decomposition, it removes interference between users, enhancing signal quality and system performance.  b. The drawbacks include high computational complexity, particularly in large-scale systems, due to the need for matrix factorization. Additionally, zero-forcing may suffer from noise amplification and signal distortion, especially in scenarios with ill-conditioned channels or high interference levels, limiting its practical applicability.  **Simulation:** BER is implemented for the proposed method.  Channel: Rayleigh channel |
| [41] | System Model: Conventional detection algorithms  a. The conventional detection algorithms were used to analyse the throughput of the framework. It was seen that the enhancement of BER is obtained at high complexity.  b. This article [41] explores the impact of 5G technology on the development of smart hospital infrastructure. It emphasizes the roles of 5G, Artificial Intelligence (AI), and the Internet of Things (IoT) in transforming healthcare delivery.  c. The paper examines various 5G features like network slicing, ultra-reliable low latency communication (uRLLC), and enhanced mobile broadband (eMBB) and how they influence smart hospital deployments.  Simulation Results: PAPR and BER of different waveforms were estimated.  Channel: Rayliegh |
| [42] | System model: 5G-IPv6 network: Enables fast and reliable data transmission.  a. The authors in [42] proposed a novel framework for utilizing 5G technology to improve healthcare monitoring. It addresses key challenges such as minimizing latency, accurately identifying health situations, and ensuring secure data sharing.  Simulation Results: The framework has been successfully tested in a prototype system for monitoring hypertensive heart disease, demonstrating high accuracy and reduced latency. |
| [43]. | System Model: Review on 5G with AI  a. The article provides a comprehensive study on features, serviceable pillars, and applications and explores how 5G is poised to fundamentally alter the healthcare landscape. However, challenges like data security, infrastructure costs, and ethical considerations require careful attention for successful implementation were discussed.  Simulation Results: No simulation was performed. |
| Proposed Work | Proposed system Mode 1: QRM-MLD-MMSE:  a, MMSE detection is a technique that minimizes the mean square error between the transmitted symbols and the detected symbols.  b. QRM-MLD-MMSE employs MMSE detection to estimate the transmitted symbols based on the received signals, taking into account the noise and interference present in the communication channel.  c. MMSE detection tends to offer improved performance over simple detection methods like maximum likelihood detection, especially in the presence of noise and interference.  Proposed System Model 2: QRM-MLD-ZF:  a. ZF detection is a technique that aims to eliminate interference from received signals by projecting the received signals onto the orthogonal complement of the interference subspace.  b. QRM-MLD-ZF uses ZF detection to recover transmitted symbols by nullifying the interference from other transmitted symbols.  c. ZF detection can be computationally intensive but offers benefits in terms of interference mitigation.  Benefits of QRM-MLD-MMSE and QRM-MLD-ZF over QRM-MLD-BF [11] include:  a. Improved Interference Rejection: MMSE and ZF detection techniques are designed to mitigate interference more effectively compared to simple beamforming techniques used in QRM-MLD-BF.  b. Better Performance in Noisy Environments: MMSE detection takes into account the noise statistics, resulting in better performance in noisy communication channels compared to beamforming alone.  c. Enhanced Signal Recovery: ZF detection, by nullifying interference, can provide better recovery of transmitted symbols compared to beamforming, especially in scenarios with significant interference.  Simulations Parameters: BER and PSD are implemented.  Channel: Rayleigh channel and Rician channel  Sub-carriers :64 and 256 |
